# Mulheres, sexismo e resistências: o declínio do patriarcado em verso e cor

**DOI:** 10.1590/0102-311XPT157125

**Published:** 2025-12-01

**Authors:** Paulo Alexandre de Souza São Bento

**Affiliations:** 1 Instituto Nacional de Saúde da Mulher, da Criança e do Adolescente Fernandes Figueira, Fundação Oswaldo Cruz, Rio de Janeiro, Brasil.

As autoras Marta Breen e Jenny Jordahl, secundadas por seu *best-seller Mulheres na Luta: 150 Anos em Busca de Liberdade, Igualdade e Sororidade* ^1^, unem-se novamente para escrever o livro, nestas páginas resenhado, intitulado *A Queda do Patriarcado: O Combate ao Machismo Através dos Séculos* ^2^. O material, publicado pela Editora Seguinte, possui tradução do norueguês de Kristin Lie Garrubo e oferece uma crítica ao patriarcado construída com objetividade e criatividade. É uma oportuna produção de memória, em linguagem simples e acessível.

A obra traz histórias que ultrapassam a dimensão do mundo das palavras. Em suas páginas coloridas, as autoras versam (ou valsam?), de modo coreografado, sobre o enfraquecimento das estruturas que sustentam as opressões de gênero, onde se encontram, de um lado, a riqueza textual e, de outro, a potente ferramenta da ilustração. Marta Breen é escritora e jornalista; Jenny Jordahl é ilustradora, cartunista e designer.

O primeiro bloco inicia contrapondo os termos “feminismo” e “patriarcado”, sendo o primeiro constituído a partir das lutas contra o segundo, definido como a predominância da autoridade do pai e, consequentemente, dos homens. Em seguida, as autoras delimitam o seu ponto de partida, considerando a dificuldade em datar a origem do fenômeno. Escolhem a Grécia Antiga e tecem, de pronto, uma análise dos vieses de gênero presentes nos discursos de filósofos, médicos, cientistas, religiosos e artistas. Homens que atribuíram às mulheres diversos predicativos, entre os quais estavam limitações de ordem física, sexual, social, intelectual, espiritual, racial, e de personalidade. Predicativos não do sujeito, mas de mulheres que foram assujeitadas e, portanto, subjugadas à matriz de sua própria opressão. A partir da ideia da natureza distinta entre os sexos, os papéis de gênero foram construídos de modo a negar às mulheres o acesso aos benefícios reservados aos homens na sociedade.

O estudo sobre uma “filosofia no feminino” ^3^, investigou, a partir de perguntas como: “por que não há mulheres filósofas?” e “por que mulheres não fizeram filosofia?”, a maneira como muitos filósofos ocidentais pensaram/explicaram a mulher. A pesquisa identificou que “*cabia aos próprios filósofos a responsabilidade relativa à divulgação de determinados estereótipos sobre a mulher*” (p. 9-10). Atribui-se a Platão e Aristóteles as teses seminais que enformaram o inconsciente filosófico sobre a condição feminina.

O segundo bloco dedica-se às mulheres insurgentes que impuseram resistências. Breen & Jordahl não economizam ao citar mais de 60 personalidades que não se apequenaram diante das opressões próprias do seu tempo, entre elas as brasileiras Bertha Lutz e Marielle Franco. Para destacar os desafios enfrentados por essas mulheres, algumas biografias são exploradas em pormenores: a demarcação de presença na vida pública, como no caso da escritora Germaine de Staël; a escolha do anonimato como estratégia para a publicação de textos, como o caso de Emily Dickinson; mulheres que se passaram por homens para conquistar espaços públicos, tais como Petra Herrera; e a delimitação do espaço privado como cárcere da mulher e os abusos gerados pelo casamento enquanto instituição, como Camilla Collett.

Vozes de um feminismo que, conforme definição de Tiburi ^4^ (p. 12), é “*o desejo por democracia radical voltada à luta por direitos daqueles que padecem sob injustiças que foram armadas sistematicamente pelo patriarcado*”. Trata-se de uma saída para as injustiças e desigualdades sociais mantidas pela “*máquina misógina patriarcal*” (p. 41), orientadora do pensamento que normatiza o nosso comportamento. Nesse sentido, é preciso reconhecer essas lutas, pois ignorá-las, segundo Adichie ^5^, seria desonesto. Afinal, foi o feminismo que trouxe à tona as especificidades e particularidades do problema de gênero.

No terceiro bloco, assenta-se pontualmente um juízo voltado para dois fenômenos: *male gaze* e *slut-shaming*, termos em inglês alcunhados pelo feminismo. Para o *male gaze* (“olhar masculino”), as autoras questionam diferentes culturas ao redor do mundo, nas quais o patriarcado impõe padrões rígidos, objetificando e secundarizando as mulheres como corpos-alvo. Denunciam, a partir das antíteses de gênero, o lugar reservado às mulheres: pureza e maldade; virgindade e vida sexual; santidade e pecado.

O *slut-shaming* é um termo que designa os estigmas referentes aos comportamentos sexuais das mulheres em sociedade. Nove personalidades são citadas pela obra, com ênfase em Charlotte Corday e Mary Wollstonecraft, que tiveram sua reputação arruinada pelo patriarcado. Mais tarde, foram os escritos de Wollstonecraft que influenciaram as expoentes do movimento feminista, como: Lucrettia Mott e Millicent Fawcett. Posteriormente, foi a vez de Alexandra Kollontai sofrer *slut-shaming* por parte de Lênin e Trótski.

O quarto e último bloco do livro aponta para uma retratação necessária − àqueles a quem se atribuiu genialidade. Resgatam a etimologia do termo *genius*, do latim “aquilo que caracterizava a natureza e a índole do homem”, ou seja, um universo de reconhecimento masculino. Para as mulheres foi reservada a invisibilidade − não se reconhece a genialidade das mulheres que foram perseguidas, torturadas e queimadas durante a Inquisição; das feministas decapitadas na guilhotina durante o século XVIII; assim como das sufragistas aprisionadas durante o século XIX.

Derradeiramente, as autoras deslocam a narrativa do passado para o presente e o futuro. Com a internet diluindo barreiras geográficas, novas gerações de feministas estão se organizando, partilhando ideias e conhecimentos. Destacam que os movimentos feministas contemporâneos têm traçado paralelos com diversas outras formas de discriminação, valorizando distintas pautas de luta por direitos, capitaneadas por aquelas que estão fora dos padrões sociais.

Assim, Breen & Jordahl, personagens de seu próprio texto, brindam à esperança no futuro e à anunciação da queda do patriarcado. O livro encerra-se como em uma peça de teatro e o *curtain* acontece com as autoras laureando, espirituosamente, os “machistas-mor” da humanidade − uma alusão à vaidade masculina e à preeminência patriarcal em ocupar postos de destaque. As cortinas se fecham após as sarcásticas menções honrosas.

Diniz & Gebara ^6^ enunciam a diversidade do feminismo através de 12 verbos da língua portuguesa. A obra aqui resenhada evoca a conjugação do verbo “lembrar”. Resgatam-se, pois, as lembranças de mulheres, seus tempos e suas histórias. Decerto, amealhar as memórias dos desfeitos, malfeitos e benfeitos constitui parte fundamental para a construção de uma nova historiografia feminista. Trata-se de “*um caminho coletivo anterior ao nosso, que guardamos muito dele e somos o que somos por causa dele*” (p. 117). Um gesto marcadamente político que possibilita tensionar o passado e mobilizar o presente para futuros possíveis − “*para o feminismo, todo testemunho de mulher é um ato de desmantelo do patriarcado*” (p. 113).

Concisamente, o material atende ao que se propõe: a crítica une-se à arte para produzir uma análise bem-humorada, própria e característica de uma história em quadrinhos (HQ). Trata-se de uma obra não capitular e organizada de modo disruptivo, tão intencional quanto a necessidade urgente de abalar as estruturas hegemônicas que as autoras denunciam. O que está em jogo não é a norma, mas a ruptura e o contradiscurso. Um estilo singular que faz do livro um convite oportuno, sagazmente construído, para refletir sobre as trajetórias de mulheres que ousaram desobedecer à perversa ordem instituída.

A relevância da publicação se alicerça como uma convocatória capaz de provocar os leitores sobre seu papel na sociedade, no que diz respeito às complexas tessituras de gênero e suas interseções, entendendo que o presente vivido também produz histórias. Uma obra orquestrada por suas maestrinas, Breen & Jordahl, que produzem versos como da canção *Pagu* (de Rita Lee e Zélia Duncan, Universal Music): “*mexo, remexo na inquisição. Só quem já morreu na fogueira sabe o que é ser carvão*”. Em paráfrase, são as autoras feministas afirmando para o mundo que “*são pau para toda obra; e que Deus (ou seriam as deusas?) dá asas às suas cobras*”. Uma leitura destinada a pessoas que desejam construir um presente comprometido com a diversidade e a justiça social.
